# Age and Upper Airway Obstruction: A Challenge to the Clinical Approach in Pediatric Patients

**DOI:** 10.3390/ijerph17103531

**Published:** 2020-05-18

**Authors:** Nosetti Luana, Zaffanello Marco, De Bernardi Francesca, Piacentini Giorgio, Roberto Giulia, Salvatore Silvia, Simoncini Daniela, Pietrobelli Angelo, Agosti Massimo

**Affiliations:** 1 Division of Pediatrics, “F. Del Ponte” Hospital, University of Insubria, 21100 Varese, Italy; luana.nosetti@uninsubria.it (N.L.); g.roberto91@icloud.com (R.G.); silvia.salvatore@uninsubria.it (S.S.); daniela_simoncini@libero.it (S.D.); 2 Department of Surgical Sciences, Dentistry, Gynecology and Pediatrics, University of Verona, 37134 Verona, Italy; marco.zaffanello@univr.it (Z.M.); giorgio.piacentini@univr.it (P.G.); 3 Department of Otorhinolaryngology, University of Insubria and ASST Sette Laghi, Ospedale di Circolo, 21100 Varese, Italy; francesca.debernardi@asst-settelaghi.it; 4 Pennington Biomedical Research Center, Baton Rouge, LA 225, USA; 5 Division of Neonatology and Neonatal Intensive Care Unit, “F. Del Ponte” Hospital, 21100 Varese, Italy; massimo.agosti@ospedale.varese.it

**Keywords:** brief resolved unexplained event, gastro-esophageal reflux, infants, laryngomalacia, obstructive sleep disordered breathing, polysomnography

## Abstract

Upper airway abnormalities increase the risk of pediatric morbidity in infants. A multidisciplinary approach to obstructive sleep apnea syndrome (OSAS) poses challenges to clinical practice. The incidence and causes of OSA are poorly studied in children under 2 years of age. To fill this gap, we performed this retrospective observational study to determine the causes of obstructive sleep apnea (OSA) in children admitted to our hospital between January 2016 and February 2018, after a brief unexplained event (BRUE) or for OSA. We reviewed the medical charts of 82 patients (39 males; BRUE n = 48; OSAS n = 34) and divided them into two age groups: < 1 year old (1–12 months; n = 59) and >1 year old (>12–24 months; n = 23). Assessment included nap polysomnography, multichannel intraluminal impedance-pH, and nasopharyngoscopy. Sleep disordered breathing was comparable between the two groups. Omega-shaped epiglottis, laryngomalacia, and nasal septum deviation were more frequent in the younger group, and nasal congestion in older group. Tonsillar and adenoidal hypertrophy was more frequent in the older group, while laryngomalacia and gastroesophageal reflux was more frequent in the younger group. Tonsil and adenoid size were associated with grade of apnea-hypopnea index severity in the older group, and laryngomalacia and gastroesophageal reflux in the younger group. The main causes of respiratory sleep disorders differ in children before or after age 1 year. Our findings have potential clinical utility for assessing the pathophysiology of obstructive sleep disordered breathing in patients less than 2 years old.

## 1. Introduction

Obstructive sleep apnea syndrome (OSAS) describes a spectrum of abnormal breathing patterns during sleep, characterized by snoring and respiratory effort secondary to increased upper airway resistance and pharyngeal collapsibility, with alterations of normal oxygenation, ventilation, and sleep architecture. Intermittent desaturation during sleep has multiorgan implications from childhood to adulthood [1,2]. The estimated frequency of OSAS is between 1% and 5% in preschool and school children [2]. 

Snoring and noisy breathing are OSAS-related symptoms during the first 2 years of life, followed by apnea, sleep movement, oral breathing, and recurrent awakenings. These symptoms are often related to micrognathia [3]. Micrognathia and body position reduce muscular tone, increase pharyngeal collapse, and reduce upper airway volumes. They are key factors in night-time sleep disorders [4]. Based on pressure transducer measurement, the main sites of upper airway obstruction are retro-palatal (52%) and retro-glossal (48%) [5]. Neck position is another important factor in airway collapsibility [6]. Restriction of the upper airways and negative pressure during inhalation are also implicated in upper airway collapse.

Obstructive sleep apnea (OSA) is a cause of apparent life-threatening events (ALTE), brief resolved unexplained events (BRUE), and sudden infant death syndrome (SIDS). A reduction in airways size due to mucosal edema and an increase in adenoid size from bacterial or viral infections have been observed in children aged 6 weeks and older [7]. Another cause of upper airway obstruction is laryngeal chemoreflex, a physiological protective mechanism that prevents inhalation of liquids. Gastroesophageal reflux (GER) and apnea may share a common relationship [8].

Nocturnal polysomnography (sleep study) is the only diagnostic technique shown to quantitate the ventilatory and sleep abnormalities associated with sleep disordered breathing (SDB), and it is currently the gold standard [2]. ALTE is not considered an event that requires PSG. Cardiorespiratory monitoring in children who snore is problematic [9]. Katz et al. distinguished different types of SDB during the first 2 years of life: periodic breathing, apnea of prematurity, and central apneas. However, OSAS has been poorly investigated in very young children [4]. 

The incidence of OSA peaks between age 2 and 8 years; a plausible explanation is the increase in the size of lymphoid tissue of the neck in this age group [10]. Importantly, however, the incidence and causes of OSAS are underinvestigated in young infants (<2 years of age) [11], even when associated with a history of ALTE/BRUE [3]. Nevertheless, early intervention is life-saving in young children with OSA or can reduce complications later on [3]. Greenfeld et al. found that the morbidity of OSAS is more severe in infants [12] and our group found that the parents of children with a history of ALTE/BRUE reported more sleep disorders than controls [13]. Furthermore, Guilleminault el al. observed that ALTE/BRUE in newborns were more prevalent in children with micrognathia, retrognathia, and bifid uvula [14], all anatomic anomalies that increase the risk of OSAS [3]. Piteo et al. reported that SDB at age 6–18 months increases the risk of hyperactivity at age 7 years [15]. 

With this study we evaluated the multidisciplinary approach to OSA in young children, and the role of pediatric sleep specialists, otolaryngologists, and gastroenterologists in particular. We reviewed the medical charts of children at the time of admission to our institution and analyzed the findings from ENT assessment, gastroenterology evaluation (GER), and PSG. There are data suggesting that children younger than 1 year have different pathophysiology of OSA and BRUE than those having more than 1 year of age, the aim of the study was to investigate characteristic and severity based on a multidisciplinary approach. 

## 2. Subjects and Methods

We performed this retrospective observational study at the Pediatric Clinic of the University of Insubria (Italy), with the collaboration of the Otorhinolaryngology and Gastroenterology Service. The protocol was approved by the Institutional Ethics Committee of the University of Insubria (n. 110/2017).

Exclusion criteria were age <1 month, central apneas at PSG, intercurrent infection, genetic syndromes, or neuromuscular disorders. Inclusion criteria were age between 1 month and 2 years, hospitalization for suspected OSAS (reported snoring, sleep apneas, oral breathing in response to nasal loading,) or after a BRUE. We assumed that a BRUE can be a manifestation of OSA in very young infants. Assessment included nap PSG, upper airway fibroscopy, and multichannel pH intraluminal impedanceometry. Children positive at PSG (Apnea-Hypopnea Index [AHI] > 1 event/h) underwent subgroup analysis.

The study population was 82 children (39 males) with a history of BRUE or OSA admitted to our institution between January 2016 and February 2018. Two age groups were formed: age <1 year (1–12 months; 72%) and age > 1 year (13–24 months; 28%).

### 2.1. Nap Polysomnography

PSG was performed by sleep recording with an E series instrument (Compumedics P/L, Melbourne, Australia). The machine records nasal flow pressure (measured with nasal cannulas), nasal flow (thermistor), chest and abdominal movement (inductive bands) (Compumedics P/L), SpO_2_ (pulse oximetry measured at a rate of 1 sample/s) and ECG (set to 500 Hz). Carbon dioxide was monitored using transcutaneous CO_2_ (TcCO_2_). Sleep staging was based on data from electroencephalogram (EEG; channels: C4-M1, C3-M2, O1-M2, O2-M1, F4-M1, F3-M2), electro-oculogram (EOG; ROC/M1, LOC/M2), and submental electromyogram (EMG). The sleep machine included video and audio recordings and a position sensor. 

Nap PSG was recorded between two baby feedings, starting not earlier than 30 min after a meal. The child lay on his back in his bed. If the recording was less than 2 h, the study continued until the next feed. 

The sleep staging criteria refer to those for 2-month-old infants; other published criteria were used for staging sleep in children <6 months [16,17]. An experienced physician assigned the sleep score (LN). The OSAS score was mild if the obstructive apnea-hypopnea index (AHI) was 1 to 5 episodes/ h; moderate if the AHI was 5–10 episodes/h, and severe if the AHI was >10 episodes/h [18]. 

### 2.2. Multichannel Intraluminal Impedance-pH

Multichannel intraluminal impedance-pH monitoring (MII-pH) was used to determine reflux. The method has been used to detect liquid, gaseous or mixed reflux, acid reflux or weakly alkaline reflux.

### 2.3. Nasopharyngoscopy

Laryngomalacia was evaluated by nasopharyngoscopy. Children with moderate–severe OSA or reported snoring, nasal loading, and oral breathing underwent direct laryngoscopy under general anesthesia to reveal airway injury [4]. Optical fiber laryngoscopy was performed, using a flexible Pentax FNL-10 RP3 rhinolaryngoscope (Pentax, Tokyo, Japan) (3.4 mm). 

### 2.4. Statistical Analysis

Statistical analysis was performed using StatView software. The Mann-Whitney test and the Kruskal-Wallis test were used to compare continuous measures and the two-way contingency test (chi-square test) to analyze frequencies. Statistical significance was set at *p* < 0.05.

## 3. Results

Table 1 presents the sample characteristics by age group: 59 infants (age 1 to 12 months) and 23 children (age 13 to 24 months). In the younger group, 19% were hospitalized for OSAS and 81% for a BRUE. In the older group, all were hospitalized for OSAS.

Table 2 presents the PSG findings for the two age groups. In the older group, the mean peripheral capillary oxygen saturation (SpO_2_) (*p* = 0.043) and the minimum SpO_2_ (%) were lower in the older group, and the AHI (events/h) was higher, but neither was significant (*p* = NS). The distribution of AHI severity showed no statistically significant difference between the two age groups (*p* = 0.067). 

Table 3 presents the overall otorhinolaryngology and gastroenterology findings by age group. The frequency of distribution of laryngeal, nasal, and turbinate abnormalities was statistically different (*p* < 0.001). In particular, omega-shaped epiglottis, laryngomalacia, and nasal septum deviation were more frequent in the younger group, and nasal congestion in the older group. Adenoid and tonsillar hypertrophy were more frequent in the older group (*p* < 0.001). GER was more common in the younger group. 

Table 4 presents the frequency (number and %) of laryngomalacia, adenoidal and tonsillar hypertrophy, GER (bold), and grade of AHI severity (mild, moderate, severe) in the total sample and the two age groups. Tonsillar and adenoidal hypertrophy and laryngomalacia were more frequent in the older group, while GER was more frequent in the younger group. 

Analysis of AHI grade (mild, moderate, severe) in relation to clinical findings (laryngomalacia, adenoidal and tonsillar hypertrophy, and GER) showed that tonsillar and adenoid size was associated with AHI severity in the older age group, while laryngomalacia and GER were associated with AHI severity in the younger age group (Table 4). 

## 4. Discussion

Obstructive sleep apnea (OSA) is a serious problem in children. OSAS in young children aged 1–23 months of age is multifactorial, and requires evaluation and treatment of the abnormalities underlying upper airway obstruction during sleep. Polysomnography is the gold standard for diagnosing OSAS in infants, and endoscopy is a useful tool for characterizing the severity of upper airway collapse [18]. Katidis et al., in their review of 159 articles on SDBs in children aged 1–23 months, underlined the importance of a multidisciplinary approach to diagnosis and treatment [18].

To our best knowledge, ours is the only study that examines OSA in very young children. In our non-syndromic patients aged between 1 and 12 months, BRUE (mainly high-risk) were the most common clinical manifestation, whereas symptoms suggesting OSA were less frequent. In patients aged 13 to 24 months, the only clinical presentation was symptoms suggesting OSAS. The AHI severity was comparable between the two age groups. Upper airway endoscopy showed a difference in the frequency of ENT anomalies: omega-shaped epiglottis, laryngomalacia, and nasal septum deviation were more frequent in the younger group, and nasal congestion in the older group. Adenoidal and tonsillar hypertrophy and severity were more prevalent in the older group. GER was often observed in the younger group. Finally, we found that tonsillar and adenoid size was associated with AHI severity in the older children, while laryngomalacia and GER were associated with AHI severity in the younger children. 

A BRUE may be the first clinical sign of OSA. One study reported that BRUE may be predictive of the development of SDB and malocclusion, highlighting the importance of long-term follow-up [19]. These results suggest that children with a history of BRUE should be evaluated for OSA at PSG. Furthermore, infants with a history of BRUE require a baseline PSG on which to base clinical recommendations. 

Data from 770 infants in the Edmonton sub-cohort of the Canadian Healthy Infant Longitudinal Study (CHILD) identified four SDB phenotypes during the first 2 years of life: no SDB, early-onset SDB (15.7%) with peak symptoms at 9 months, late-onset SDB (14.2%) with peak symptoms at 18 months, and persistent SDB (5.3%) with symptoms from 3 to 24 months [3]. Rhinitis was associated with all three SDB symptom trajectories [20]. During the first 2 years of life, the causes of OSAS are mainly anomalies of upper airway anatomy (laryngomalacia, choanal atresia, macroglossia, and craniofacial malformations in syndromic patients). Differently, upper respiratory tract obstruction secondary to hypertrophy of the lymphoid tissue of the neck was reported to begin at 2 years of age [10]. Consistent with our findings, an early survey showed that laryngomalacia and omega-shaped epiglottis were associated with OSAS in infants aged < 1 year- [21]. 

Multichannel intraluminal impedance-pH monitoring (MII-pH) allows for the detection of liquid, gas or mixed reflux, acidic or weakly alkaline reflux [22,23]. Recent data have shown an association between atypical (extra-esophageal) symptoms and non-acid reflux. This finding suggests that non-acid reflux may be significant in childhood. Non-acid reflux accounted for 48% of total reflux in 25 infants with apnea or a history of BRUE [24] and apnea of prematurity [25].

According to a NICE report, signs of GER are less frequent in infants aged 1–2-years [26] because they tend to disappear physiologically after 1 year of age [26]. In brief, upper airway inflammation, laryngitis, reduced laryngo-pharyngeal reflex, increased secretions resulting in hypopharyngeal obstruction, tracheal aspiration, laryngospasm, and apnea/desaturation are the main pathophysiological mechanisms. Laryngeal, rhinopharyngeal, and oropharyngeal alterations are known to play a significant role in nasal obstruction and respiratory events. Presently, there is no convincing evidence for GER in the pathophysiology of SDB in infants aged <12 months, although a relationship between OSAS and GER has been investigated and suggested [27,28]. Menon and colleagues reported an increased frequency of apnea in infants with regurgitation, but this was not related to GER [27,29]. Recently, Kamal et al. reported that newborns with GER had early and late OSA [20]. Our data suggest a relationship between GER and OSA, although we cannot exclude that GER may be an incidental finding physiologically present in this cohort of young infants. Laryngeal anomalies also present in this cohort might explain the observed OSA; however, we cannot ignore the frequency of GER in this cohort of young infants with OSA. 

A limitation of this study was the use of nap PSG, instead of overnight full PSG. Some patients may have had falsely negative nap PSG. When the parameters of the nap study are abnormal, however, the probability of OSA is reported to be elevated [30]. Another limitation is that our cohort is a subgroup analysis. Moreover, we did not present the central apneas and NREM and REM sleep stages, because it is almost impossible to compare young infants versus older infants, particularly by using nap-polysomnography recordings. Finally, the low numbers in each subgroup is a limitation and some of the negative findings were likely due to these low numbers.

Summarizing, the main causative factors of OSA in the young group (age < 1 year) were laryngomalacia and GER, while tonsillar and adenoidal hypertrophy were more prominent in the older group (age 1–2 years). Laryngomalacia is commonly associated with GER [31]. In most cases, the natural history of laryngomalacia is benign, and patients recover spontaneously with time. In 9–29% of infants, laryngomalacia may result in moderate-severe upper airway obstruction [31]. Adenoid and tonsillar hyperplasia should be considered in children aged 6 months and older, because it is a frequent cause of OSAS. [32] In a study involving 28 children (age 1.3–1.8 years), the severity of obstructive AHI (7.5–28.3 events/h) was in line with our findings. The majority of these children had adenoid hypertrophy and tonsillar hyperplasia [11]; however, tonsil/adenoid size does not necessarily predict the grade of AHI severity [33]. 

Our data indicate a difference in the main causes of OSA between the two age groups. Increased tonsil and adenoid size were associated with OSA severity in the older children and laryngomalacia and GER were associated with OSA severity in the younger children. Our results have potential clinical utility in evaluating the pathophysiology of OSA in young infants. A multidisciplinary approach may provide the best strategy to manage young patients with upper airway obstruction. 

## Figures and Tables

**Table 1:** Characteristics of the sample based on clinical diagnosis at admission and age.

Characteristic	Age 1 Month–12 Months	Age 13 Months–24 Months
Total-No. (%)	59 (72)	23 (28)
**Diagnosis at Admission**		
OSAS	11 (19)	23 (100)
BRUE	48 (81)	-
Low Risk	12 (25)	-
High Risk	36 (75)	-

BRUE denotes brief resolved unexplained events; OSAS: obstructive sleep apnea syndrome.

**Table 2:** Polysomnography findings.

Sleep Respiratory Parameters	Total (n = 82)	Age 1–12 Months (n = 59)	Age 13–24 Months (n = 23)	Mann-Whitney Test *p*-Value
Mean SpO_2_ (%)	97.8 ± 1.2	98.0 ± 1.0	97.0 ± 1.5	0.043
Min SpO_2_ (%)	89.0 ± 6.3	89.0 ± 5.0	87.0 ± 6.5	NS
AHI (events/h)	3.8 ± 7.1	3.6 ± 6.3	5.7 ± 7.7	NS
**AHI Severity**	**No. (%)**	**No. (%)**	**No. (%)**	**Two-Way Contingency Table** **Chi Square test (*p*-value)**
Mild	42 (51.2)	33 (55.9)	10 (43.5)	
Moderate	21 (25.6)	16 (27.1)	6 (26.1)	
Severe	19 (23.2)	10 (17.0)	7 (30.4)	5.354 (*p* = 0.067)

SpO_2_ denotes oxygen saturation; AHI: apnea-hypopnea index.

**Table 3:** Diagnostic findings of otorhinolaryngology and gastroenterology.

Diagnosis	Total No. (%)	Age 1–12 Months No. (%)	Age 13–24 Months No. (%)	Two-Way Contingency Table Chi Square Test (*p*-Value)
Omega-Shaped Epiglottis	12 (14.6)	11 (18.6)	1 (4.3)	
Laryngomalacia	13 (15.9)	12 (20.3)	1 (4.3)	
Choanal Atresia	1 (1.2)	0 (0.0)	1 (4.3)	
Nasal Congestion	10 (12.2)	6 (10.2)	4 (17.4)	
Nasal Septum Deviation	3 (3.7)	3 (5.1)	0 (0.0)	
Turbinate Hypertrophy	10 (12.2)	7 (11.9)	3 (13.0)	27.088 (*p* < 0.001)
Gastroesophageal Reflux	34 (41.5)	30 (50.8)	4 (17.4)	
Adenoid Hypertrophy	33 (38.0)	13 (22.0)	20 (87.0)	
Grade 1	11 (33.3)	9 (69.2)	2 (10)	26.7
Grade 2	9 (27.3)	2 (15.4)	7 (35)	
Grade 3	9 (27.3)	1 (7.7)	8 (40)	
Grade 4	4 (12.1)	1 (7.7)	3 (15)	76.092 (*p* < 0.001)
Tonsillar Hypertrophy	27 (32.9)	10 (16.9)	17 (73.9)	
Grade 1	11	6 (60)	5 (29.4)	
Grade 2	9	3 (30)	6 (35.3)	
Grade 3	6	1 (10)	5 (29.4)	
Grade 4	1	0 (0)	1 (5.9)	26.356 (*p* < 0.001)

**Table 4:** Frequency (number and percentage) of laryngomalacia, adenoid, and tonsillar hypertrophy, and gastroesophageal reflux in the total sample (bold) and subgroups, and grade of apnea-hypopnea index severity (mild, moderate, severe).

ENT Findings and AHI Severity	Total (n = 82)	Age 1–12 Months (n = 59)	Age 13–24 Months (n = 23)	Two-Way Contingency Table Chi Square Test (*p*-Value)
Laryngomalacia-No. (%)	12 (14.6)	11 (18.6)	1 (4.3)	
Mild AHI–No. (% Mild/Total Mild)	7 (16.7)	6 (18.2)	1 (10)	
Moderate AHI–No. (% Moderate /Total Moderate)	0 (0)	0 (0)	0 (0)	
Severe AHI–No. (% Severe /Total Severe)	5 (26.3)	5 (50.0)	0 (0)	NC
Adenoid Hypertrophy-No. (%)	33 (40.2)	13 (22.0)	20 (87.0)	
Mild AHI–No. (% Mild /Total Mild)	12 (28.6)	7 (21.2)	7 (70.0)	
Moderate AHI–No. (% Moderate /Total Moderate)	9 (42.9)	2 (12.5)	6 (100)	
Severe AHI–No. (% Severe /Total Severe)	7 (36.8)	4 (40.0)	4 (57.1)	25.610 (<0.001)
Tonsillar Hypertrophy-No. (%)	27 (32.9)	10 (16.9)	17 (73.9)	
Mild AHI–No. (% Mild /Total Mild)	10 (23.8)	6 (18.2)	5 (50)	
Moderate AHI–No. (% Moderate /Total Moderate)	8 (38.1)	2 (12.5)	6 (100)	
Severe AHI–no. (% Severe /Total Severe)	6 (32.6)	3 (30.0)	3 (42.9)	22.315 (*p* < 0.001)
Gastroesophageal Reflux-No. (%)	34 (41.5)	30 (50.8)	5 (21.7)	
Mild AHI (% Mild /Total Mild)	19 (45.2)	17 (51.5)	2 (20)	
Moderate AHI–No. (% Moderate /Total Moderate)	11 (52.4)	9 (56.3)	2 (20)	
Severe AHI–No. (% Severe /Total Severe)	5 (26.3)	4 (40.0)	1 (10)	1.047 (*p* = NS)
Total				79.889 (*p* < 0.001)

AHI denotes apnea-hypopnea index; NC: not computable.

## Abbreviations

ALTE	acute life-threatening event
BRUE	brief unexplained event
GER	gastroesophageal reflux
ORL	otorhinolaryngology
OSA	obstructive sleep apnea
PSG	polysomnography
SDB	sleep disordered breathing
